# Secure Hierarchical Asynchronous Federated Learning with Shuffle Model and Mask–DP

**DOI:** 10.3390/s26020617

**Published:** 2026-01-16

**Authors:** Yonghui Chen, Daxiang Ai, Linglong Yan

**Affiliations:** 1 School of Computer Science, Hubei University of Technology, No. 28 Nanli Road, Hongshan District, Wuhan 430068, China; 102301194@hbut.edu.cn (D.A.); m19172877920@163.com (L.Y.); 2 Hubei Provincial Engineering Research Center for Digital & Intelligent Manufacturing Technologies and Applications, No. 28 Nanli Road, Hongshan District, Wuhan 430068, China; 3 Hubei Provincial Key Laboratory of Green Intelligent Computing Power Network, No. 28 Nanli Road, Hongshan District, Wuhan 430068, China

**Keywords:** federated learning, differential privacy, secure aggregation, consensus mechanism, shuffle model

## Abstract

Hierarchical asynchronous federated learning (HAFL) accommodates more real networking and ensures practical communications and efficient aggregations. However, existing HAFL schemes still face challenges in balancing privacy-preserving and robustness. Malicious training nodes may infer the privacy of other training nodes or poison the global model, thereby damaging the system’s robustness. To address these issues, we propose a secure hierarchical asynchronous federated learning (SHAFL) framework. SHAFL organizes training nodes into multiple groups based on their respective gateways. Within each group, the training nodes prevent inference attacks from the gateways and committee nodes via a mask–DP exchange protocol and employ homomorphic encryption (HE) to prevent collusion attacks from other training nodes. Compared with conventional solutions, SHAFL uses noise that can be eliminated to reduce the impact of noise on the global model’s performance, while employing a shuffle model and subsampling to enhance the local model’s privacy-preserving level. At global model aggregation, SHAFL considers both model accuracy and communication delay, effectively reducing the impact of malicious and stale models on system performance. Theoretical analysis and experimental evaluations demonstrate that SHAFL outperforms state-of-the-art solutions in terms of convergence, security, robustness, and privacy-preserving capabilities.

## 1. Introduction

Hierarchical asynchronous federated learning (HAFL) has been widely applied and studied across various academic and industrial scenarios [1,2,3,4,5]. HAFL can adapt to more realistic networking systems with hierarchical structures and be compatible with heterogeneous training nodes through an asynchronous update mechanism. Typical applications include the Internet of Vehicles (IoV) [6,7,8] and the Internet of Things (IoT) [9,10,11,12]. However, HAFL still faces FL-specific security issues, including single-point failure, data privacy, and Byzantine fault tolerance. Attackers may conduct inference attacks to reconstruct the training nodes’ datasets from their updated models [13,14]. Malicious nodes may launch Byzantine attacks to compromise system robustness by poisoning the model [15,16].

Centralized federated learning (FL) approaches always suffer from a single point of failure and untrusted aggregation [17,18]. Owing to features such as decentralization, immutability, traceability, and consensus mechanisms, Blockchain-based technologies offer effective solutions [6,18,19]. They use Blockchain to store the global model, computational metadata, and other relevant data generated during the training process, ensuring transparency, traceability, and tamper resistance. However, Blockchain-based FL still faces privacy-preserving problems, e.g., membership inference attacks [13,20], model inversion attacks [14], Byzantine attacks, e.g., poisoning the models [15,16], and label flipping [21,22].

Differential privacy (DP) has been widely used for privacy in FL [23,24,25]. Compared to homomorphic encryption (HE) [26,27,28] and secure multi-party computation (SMC) [29,30,31], DP has low computational overhead and is more suitable for multiple iterations of computation [32]. Central differential privacy (CDP) [33] inputs calibrated noise into the global model via a central server that aggregates the model. Local differential privacy (LDP) [34] eliminates the dependence on a trusted central server and allows each training node to add noise to the uploaded model. However, the accumulated noise may degrade the performance of the global model. Yuan et al. [35] proposed an adaptive perturbation scheme that adjusts the variance of the perturbation online to reduce the performance degradation. Sun et al. [24] combined LDP with a shuffle model to reduce noise variance and enlarge the privacy budget. However, they can only reduce, but not eliminate, the impact of noise.

To suppress Byzantine attacks, e.g., additive noise (AN) [36,37], A Little Is Enough (ALIE) [22], inner product manipulation (IPM) [38], sign flipping (SF) [39,40], and label flipping (LF) [21,41], numerous robust aggregation algorithms have been proposed, e.g., Euclidean distance-based methods [42,43,44,45], cosine similarity-based approaches [46,47], and median/mean-based statistical techniques [48]. These algorithms distinguished between honest and malicious nodes by leveraging geometric distances or statistical features in high-dimensional spaces.

However, in LDP-based HAFL, the geometric distances or statistical characteristics are disturbed by noise, making it hard to distinguish malicious and delay models. Designing an HAFL system that simultaneously ensures privacy-preserving and Byzantine robustness remains hard.

This study proposes a secure hierarchical asynchronous federated learning (SHAFL) framework that ensures both privacy preservation and Byzantine robustness. Our contributions are summarized as follows:

- SHAFL proposes a decentralized mask exchange protocol that uses eliminable noise to prevent the gateway from compromising the privacy of the training node and to reduce the impact of noise on global model performance. Based on HE, it prevents N−1 collusion attacks among training nodes.
- The SHAFL scheme introduces a novel mechanism for continuous layer subsampling and dummy-layer padding. Combining continuous-layer subsampling, dummy-layer padding, and a shuffle model, SHAFL enhances the privacy-preserving capability of local models during the server aggregation phase.
- SHAFL designs a secure aggregation scheme that leverages the upload model’s test accuracy to mitigate the impact of malicious nodes on system robustness.
- With an eliminable noise, SHAFL reduces the damage to system robustness caused by node offline before model shuffling in groups.
- Experiments on the MNIST, CIFAR-10, and Heart Disease datasets validate the privacy, convergence, and robustness of the proposed SHAFL.

The remainder of this study is organized as follows: Section 2 analyzes the related work; Section 3 discusses the system model; Section 4 presents the proposed SHAFL framework; Section 5 and Section 6 discuss the convergence and security of the proposed SHAFL; Section 7 presents an experimental analysis of the proposed SHAFL; and Section 8 is the conclusion.

## 2. Related Work

Xie et al. [49] proposed an asynchronous federated optimization algorithm (FedAsync) addressing the straggler issue. Miao et al. [50] proposed a time-weighted asynchronous PPFL that integrates stale models. Wu et al. [51] designed an aggregation method to control asynchronous aggregation errors. Chen et al. [52] proposed an adaptive semi-asynchronous federated learning (ASAFL) approach to balance learning latency and accuracy. However, the distributed architecture of FL makes it susceptible to privacy-preserving issues [13,14] and Byzantine attacks [22,36,37].

There are three typical privacy protection methods in FL: HE [26,27], DP [18,32,53,54], and SMC [29,30,31]. Compared with DP and SMC, HE-based methods exhibit higher computational complexity and overly conservative safety assumptions. For example, Yang et al. [26] proposed a secure FL scheme that prevents privacy attacks from external attackers and half-honest servers without requiring a shared homomorphic key. It can not defend against internal attacks from training nodes that share homomorphic keys. Miao et al. [27] proposed a privacy-preserving and Byzantine-robust FL framework with a fully homomorphic encryption (FHE) algorithm CKKS, assuming a trusted verifier. DP is widely used to preserve privacy in FL due to its quantifiable privacy loss and low computational overhead [18,32,53,54]. Wei et al. [53] proposed a Gaussian–DP-based privacy-preserving FL scheme. Jiang et al. [54] proposed a Laplace–DP-based algorithm to improve performance. Yan et al. [18] proposed a Laplace–DP-based asynchronous FL scheme for an IoT system, while analyzing the dropout tolerance of DP-based FL. However, the noise introduced by DP inherently degrades the model’s accuracy and utility and requires a larger privacy budget. In theory, Mask-based SMC schemes can eliminate the effects of noise. However, security concerns arise in the generation and aggregation of the mask/noise. For example, Feng et al. [29] proposed a Blockchain-enabled, horizontally decentralized FL with a mask that may be generated by a malicious node. Hiroki et al. [30] proposed a mask-based decentralized FL scheme; however, it cannot protect against collusion attacks. Shen et al. [31] proposed a LiPFed scheme in which each training node generates its own masks, thereby eliminating reliance on intermediate nodes for security. However, the divided model may result in insecure aggregation. Moreover, if the aggregation node cannot obtain all the noisy models, the mask/noise cannot be eliminated. In our proposed scheme, we introduce a mask-DP exchange protocol that, in theory, eliminates noise and improves performance when used with PBFT.

To address Byzantine attacks, it is necessary to distinguish between honest and malicious nodes using updated models [55,56]. With a consortium Blockchain, Yan et al. [18] adopt a Practical Byzantine Fault Tolerance (PBFT) protocol to ensure the credibility of aggregated results. Furthermore, Xu et al. [57] proposed a semi-asynchronous aggregation scheme resisting poisoning attacks, backdoor attacks, and Distributed Denial of Service (DDoS) attacks. Zhang et al. [56] proposed a robust and secure framework for FL with verifiable DP noise. However, their work is discussed in the context of synchronous FL but ignores the impact of asynchronous FL, particularly the effect of noise on the model accuracy of PBFT.

In addition, privacy amplification mechanisms, e.g., shuffler [58,59], subsampling [60], and dummy points [24], are introduced into FL to increase the privacy budget while reducing noise. The shuffling mechanism disrupts the correlation between the uploaded local models and the training nodes to enhance the LDP with anonymity [58,59,61]. Using the subsampling and dummy point algorithms, Sun et al. [24] proposed a privacy-enhancing DP-based FL, which amplified the privacy-preserving level of LDP at the aggregation stage. These methods can reduce the impact of DP noise. In our proposed scheme, we introduce a shuffling mechanism for asynchronous environments, reducing the impact of mask noise leakage on PBFT.

## 3. System Model

This section introduces the Blockchain-based hierarchical asynchronous federated learning, threat model, and privacy-preserving mechanism adopted by the SHAFL framework.

### 3.1. Blockchain-Based Hierarchical Asynchronous Federated Learning

**Figure 1:**
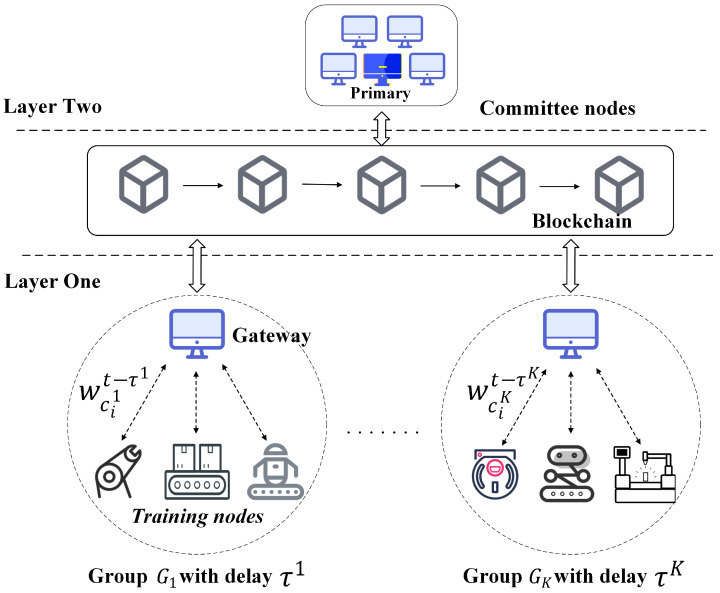
The structure of HAFL.

Shown in Figure 1, our proposed SHAFL framework considers a Blockchain-based scenario that consists of two layers; In the first layer of the SHAFL framework, the training nodes have *K* groups $\left\{G_{o}\right\}$, each group has a header node called gateway $y_{o}$, and the size of group $G_{o}$ is $s_{o}$. In group $G_{o}$, each training node $c_{i}^{o}\in G_{o}$ has a dataset $D_{c_{i}^{o}}\in D$. The basic FL [62] is

(1) $$F\left(w\right)=\sum_{c_{i}^{o}\in C}\rho_{c_{i}^{o}}F_{c_{i}^{o}}\left(w\right)$$

(2) $$F_{c_{i}^{o}}\left(w\right)=\sum_{\xi \in D_{c_{i}^{o}}}\rho_{\xi }f\left(w,\xi \right)$$

where F(w) is the task objective function, $F_{c_{i}^{o}}$ is the objective function of $c_{i}^{o}$, $\rho_{c_{i}^{o}}$ and $\rho_{\xi }$ are weights of model aggregation, and f(w,ξ) is the loss function at $c_{i}^{o}$.

In the first layer of the SHAFL framework, in turn t≤T, each training node $c_{i}^{o}$ first receives a global model $w^{t-1}$ from Blockchain; then, locally and iteratively trains $w^{t-1}$ with $D_{c_{i}^{o}}$, and outputs $w_{c_{i}^{o}}^{t-\tau^{o}}$: $w_{c_{i}^{o}}^{t-\tau^{o}}\equiv w_{c_{i}^{o}}^{t-\tau^{o},H}$, after *H* iterations. In iteration h≤H, the local update is

(3) $$w_{c_{i}^{o}}^{t-\tau^{o},h}=w_{c_{i}^{o}}^{t-\tau^{o},h-1}-\gamma_{c_{i}^{o}}g_{c_{i}^{o}}^{t-\tau^{o},h-1}\left(w_{c_{i}^{o}}^{t-\tau^{o},h-1},D_{c_{i}^{o}}\right)$$

(4) $$g_{c_{i}^{o}}^{t-\tau^{o},h}\left(w_{c_{i}^{o}}^{t-\tau^{o},h},D_{c_{i}^{o}}\right)=\frac{1}{|D_{c_{i}^{o}}|}\sum_{\xi \in D_{c_{i}^{o}}}▽f\left(w_{c_{i}}^{t-\tau^{o},h},\xi \right)$$

where $w_{c_{i}^{o}}^{t-\tau^{o}}$ is the local update, $\tau^{o}$ is the delay of group $G_{o}$ and gateway $y_{o}$, and $t-\tau^{o}$ is the start time of local training replacing synchronous tempo *t*; $\gamma_{c_{i}^{o}}$ is the learning rate.

It assumes that all local training within a group is synchronous, meaning that all τ in a group are the same and are marked with $\tau^{o}$. After collecting all local updates, the gateway $y_{o}$ obtains $w_{y_{o}}^{t-\tau^{o}}$:

(5) $$w_{y_{o}}^{t-\tau^{o}}=\frac{\sum_{c_{i}^{o}\in G_{o}}w_{c_{i}^{o}}^{t-\tau^{o}}}{s_{o}}$$

**Figure 2:**
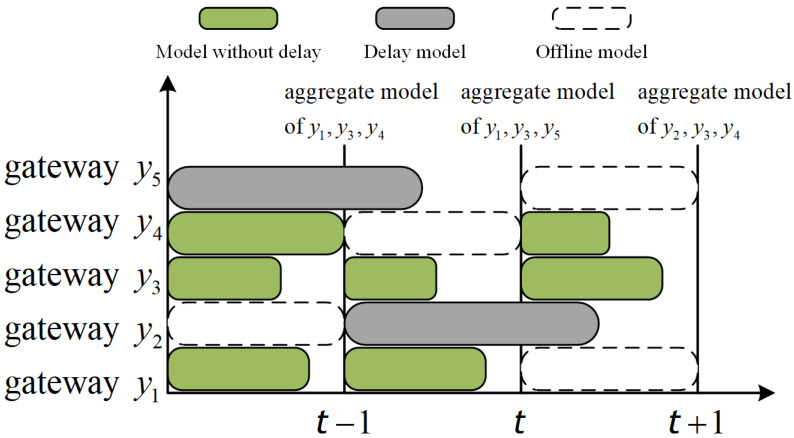
Time workflow of asynchronous update mechanism in SHAFL framework.

In the second layer of the SHAFL framework, all gateways can upload their updates to the Blockchain asynchronously, which means the primary committee node allows the gateways to have different delays $\tau^{o}$. After a period, the primary committee node downloads the updates from the Blockchain and aggregates the global model as [1]

(6) $$w^{t}=\left(1-\alpha \right)w^{t-1}+\alpha \frac{\sum_{o=1}^{\eta_{t}}\rho_{y_{o}}w_{y_{o}}^{t-\tau^{o}}}{\sum_{o=1}^{\eta_{t}}\rho_{y_{o}}}$$

where α∈(0,1) is the hyperparameter weight of global update, $\rho_{y_{o}}$ is the weight of local update $w_{y_{o}}^{t-\tau^{o}}$, and $\eta_{t}$ is the number of local updates uploaded in turn *t*. Figure 2 shows the asynchronous time workflow of the SHAFL framework.

### 3.2. Threat Model

In this study, we assume that a gateway can be honest but curious, and a training/committee node might be potentially malicious. The potential threats caused by training nodes, gateways, and committee nodes are shown as follows.

- Training nodes: They try to extract other training nodes’ local data as much as possible from local updates, via launching inference attacks [13,14] and data reconstruction attacks [63,64,65]. Malicious training clients may engage in data poisoning or upload maliciously crafted local updates [66], which can lead to a degradation of the global model’s accuracy.
- Gateways: They follow predefined protocols and submit correct intermediate results. However, they are curious about the sensitive information contained in training nodes and may attempt to infer the training nodes’ private data, resulting in data leakage.
- Committee nodes: Malicious committee nodes may discard local updates from gateways or release a malicious global model, thus compromising the robustness of the system.
- collusion attacks: Malicious training nodes may collude to obtain the private model of the target node, such as attempting to remove the noise added to the target model. Furthermore, malicious training nodes could collude with gateways, or gateways could collude with malicious committee nodes to attack the training nodes’ privacy.

### 3.3. Privacy Preserving Mechanism

To tackle the privacy-preserving issues, the SHAFL framework introduces an LDP-based shuffle model, a mask–DP exchange protocol, and Paillier homomorphic encryption.

#### 3.3.1. LDP Mechanism

Unlike CDP, an LDP-based FL allows the training node to add noise to the model locally to achieve decentralized privacy-preserving [32], which has no reliance on a trusted server.

**Definition 1**  ($\left(\epsilon_{0},\delta_{0}\right)$-LDP [32])**.** *A randomized algorithm M:D→R satisfies $\left(\epsilon_{0},\delta_{0}\right)$-LDP if for any two adjacent datasets $d,d^{\prime }\in D$ and for any subset of outputs S⊆R, it holds that*

(7) $$Pr\left[M\left(d\right)\in S\right]\leq e^{\epsilon_{0}}Pr\left[M\left(d^{\prime }\right)\in S\right]+\delta_{0}$$

Gaussian mechanism extracts random noise from the Gaussian distribution and adds noise to the query function to satisfy (ϵ,δ)-DP.

**Definition 2**  (Gaussian Mechanism [32])**.** *For a given query function f with sensitivity ${▵}_{2}f$. The randomized algorithm $M=f\left(D\right)+N\left(0,\sigma^{2}\right)$ satisfies (ϵ,δ)-DP if*

(8) $$\sigma \geq \frac{{▵}_{2}f}{\epsilon }\log\left(2\ln\left(1.25/\delta \right)\right)$$

*where $N(0,\sigma^{2})$ is a Gaussian distribution with mean 0 and covariance $\sigma^{2}$, and ${▵}_{2}f$ is $l_{2}$ sensitivity of query function f.*

#### 3.3.2. LDP-Based Shuffle Model

**Figure 3:**
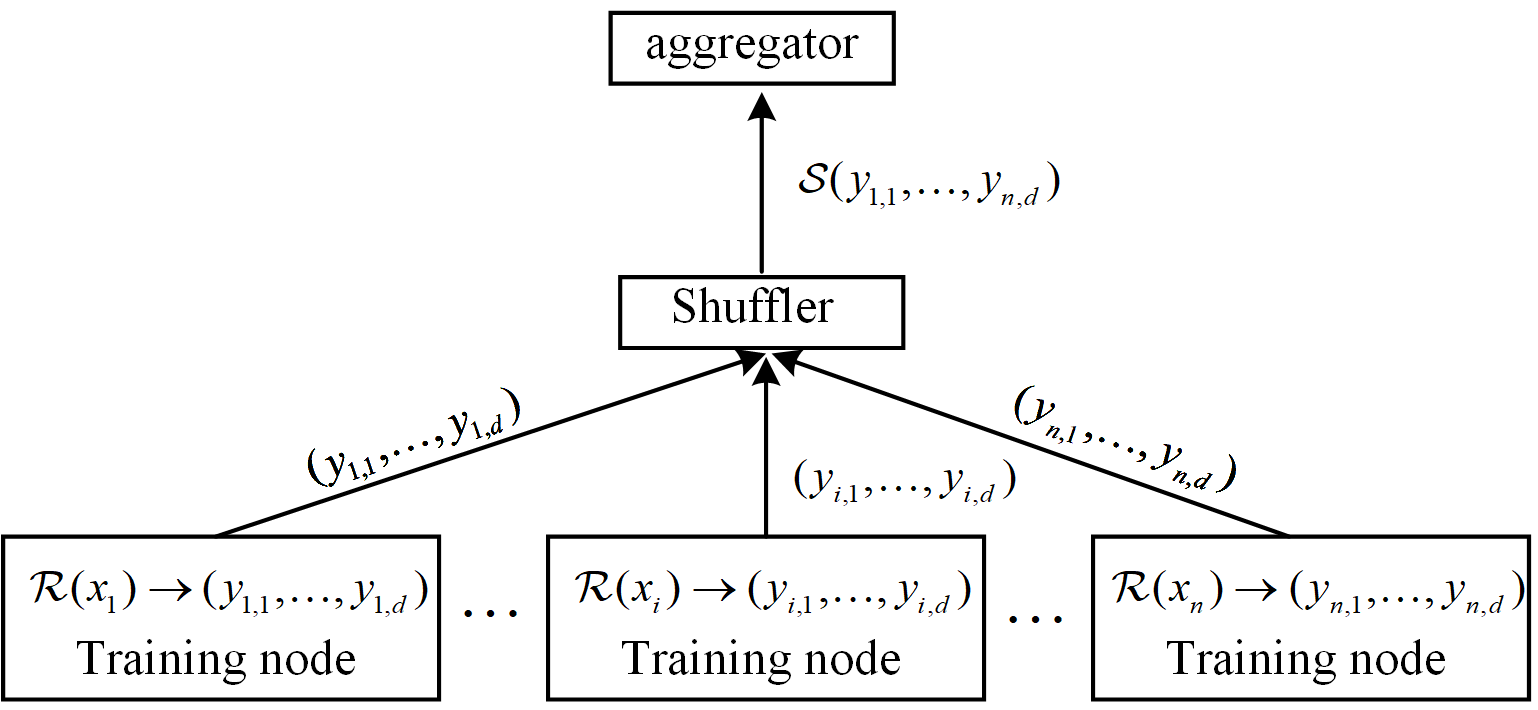
Shuffle model.

The shuffle model disrupts the correlation between the local model and the training nodes through a confusion mechanism to provide anonymity to the local model [59,61]. A LDP-based shuffle model further enhances the privacy-preserving and anonymity [58,67]. The LDP shuffle model is shown in Figure 3 and defined as follows.

**Definition 3**  (LDP-based shuffle model [24])**.** *A randomized mechanism M is an LDP shuffle model if it includes three components: encoder R, shuffler S, and analyzer A [24]. Considering that the shuffler (gateway) takes n training nodes’ upload in group $G_{o}$:*

- *Encoder $R:X\to Y^{d}$ is a randomized algorithm that runs on the training nodes’ side and converts local data $x_{i}\in X$ into d messages.*
- *Shuffler $S:{\left(Y^{d}\right)}^{n}\to {\hat{Y}}^{dn}$ collects the messages uploaded by n training nodes and processes the messages into a random permutation.*
- *Aggregator $A:{\hat{Y}}^{dn}\to Z$ aggregates the random permutation uploaded by training nodes to generate a model.*

*In summary, the shuffle DP can be denoted as*

(9) $$\begin{array}{cc} M_{y_{o}} & ≜A\circ S\circ R(X)\\ \; & =A(S\left(R\left(x_{1}\right),\ldots ,R\left(x_{n}\right)\right))\\ \; & =A(S\left(y_{1,1},\ldots ,y_{1,d},\ldots ,y_{n,1},\ldots ,y_{n,d}\right))\\ \; & =A(\underset{n}{\underset{︸}{y_{1,1},\ldots ,y_{z_{1},1}}},y_{1,2},\ldots ,y_{z_{2},2},\ldots ,y_{1,d},\ldots ,y_{z_{3},d})\\ \; & =Z \end{array}$$

*where $M_{y_{o}}$ is the privacy-preserving mechanism, d is the number of messages, $z_{1},z_{2},z_{3}$ are random numbers, and Z is the uploaded model of the shuffler (gateway). Encoder R satisfies $\left(\epsilon_{0},\delta_{0}\right)$-LDP.*

#### 3.3.3. Mask–DP Exchange Protocol

An eliminable noise [68], mask π, is generated through the Gaussian mechanism. In turn *t*, the mask exchange protocol is defined as detailed in [30]. The process is

- Input the number of training nodes *n*, the number of exchange noises m,1<m<n, and a set of privacy budgets $\left\{\epsilon_{c_{i}}\right\}$.
- Each $c_{i}$ generates mask $\pi_{c_{i}}^{t}$ based on $\left\{\epsilon_{c_{i}}\right\}$ and receives mask $\left\{\pi_{c_{j}}^{t}\right\}$ from $J=\{c_{j}\},j=i+1,\ldots ,i+m\;\mathrm{mod}\;n$.
- After the exchange step, each $c_{i}$ aggregate received masks $\pi_{i}^{t}=\sum_{j\in J}\pi_{c_{j}}^{t}$.
- Each training node $c_{i}$ generates *m* multi-masks as follows:
  (10) $$M_{i,k}^{t}=\left\{\begin{array}{cc} w_{c_{i}}^{t}-\pi_{i}^{t}, & if\;k=0\\ \pi_{c_{i}}^{t}, & if\;k=1,2,3,\ldots ,m \end{array}\right.$$
- Each $c_{i}$ sends *m* multi-masks to gateways.

**Figure 4:**
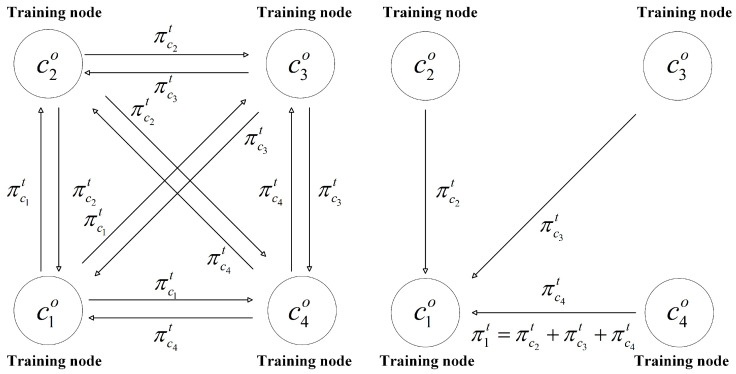
Mask–DP exchange protocol when n=4 and m=3.

The local update $w_{i}^{t}-\pi_{i}^{t}$ satisfies (ϵ,δ)-LDP. In a group $G_{o}$, $\sum_{i=1}^{n}\sum_{k=0}^{m}M_{i,k}^{t}=\sum_{i=1}^{n}w_{c_{i}}^{t}$. The server can aggregate the global model without adding perturbation. Shown in Figure 4, in a group $G_{o}$, the number of training nodes is *n*, e.g., n=4, and the number of noises to be exchanged is m=n−1=3. Each training node generates noise $\left\{\pi_{c_{1}}^{t},\pi_{c_{2}}^{t},\pi_{c_{3}}^{t},\pi_{c_{4}}^{t}\right\}$ based on its privacy budget $\left\{\epsilon_{c_{1}},\epsilon_{c_{2}},\epsilon_{c_{3}},\epsilon_{c_{4}}\right\}$. Following the mask exchange protocol, *m* multi-mask messages $\left\{M_{1,0}^{t},\ldots ,M_{4,3}^{t}\right\}$ are generated and transmitted to the gateway. The gateway then performs pre-aggregation as follows:

(11) $$\begin{array}{cc} \sum_{i=1}^{4}\sum_{k=0}^{3}M_{i,k}^{t}\; & =M_{1,0}^{t}+M_{1,1}^{t}+M_{1,2}^{t}+M_{1,3}^{t}+\ldots +M_{4,0}^{t}+M_{4,1}^{t}+M_{4,2}^{t}+M_{4,3}^{t}\\ \; & =w_{c_{1}}^{t}-\pi_{1}^{t}+\pi_{c_{1}}^{t}+\pi_{c_{1}}^{t}+\pi_{c_{1}}^{t}+\ldots +w_{c_{4}}^{t}-\pi_{4}^{t}+\pi_{c_{4}}^{t}+\pi_{c_{4}}^{t}+\pi_{c_{4}}^{t}\\ \; & =w_{c_{1}}^{t}-\pi_{c_{2}}^{t}-\pi_{c_{3}}^{t}-\pi_{c_{4}}^{t}+3\pi_{c_{1}}^{t}+\ldots w_{c_{4}}^{t}-\pi_{c_{1}}^{t}-\pi_{c_{2}}^{t}-\pi_{c_{3}}^{t}+3\pi_{c_{4}}^{t}\\ \; & =w_{c_{1}}^{t}+w_{c_{2}}^{t}+w_{c_{3}}^{t}+w_{c_{4}}^{t}\\ \; & =\sum_{i=1}^{4}w_{c_{i}}^{t} \end{array}$$

where $w_{c_{i}}^{t}$ denotes the local update of training node $c_{i}$. After pre-aggregation, the noise $\left\{\pi_{c_{1}}^{t},\pi_{c_{2}}^{t},\pi_{c_{3}}^{t},\pi_{c_{4}}^{t}\right\}$ is eliminated. Therefore, the server can aggregate a global model without perturbation.

#### 3.3.4. Paillier Homomorphic Encryption

Our scheme is based on Paillier homomorphic encryption (PHE) [69], which is an additive homomorphic encryption scheme. It consists of three algorithms.

- Key Generation: Select two large prime numbers, *p* and *q*. Calculate n=pq and λ=lcm(p−1,q−1); lcm(·) denotes the least common multiple. Randomly select $g\in Z_{n^{2}}^{*}$ satisfying $gcd(L\left(g^{\lambda }\;\mathrm{mod}\;n^{2}\right),n)=1$; gcd(·) denotes the greatest common divisor, $L\left(x\right)=\frac{x-1}{n}$. Calculate $\mu ={\left(L\left(g^{\lambda }\;\mathrm{mod}\;n^{2}\right)\right)}^{-1}\;\mathrm{mod}\;n$. Output the public key PK=(n,g) and keep the private key SK=(λ,μ).
- Encryption: Input a plain text $m\in Z_{n}$ and select a random number $r\in Z_{n}^{*}$. Output the cipher text $c=Enc\left(m\right)=g^{m}\cdot r^{n}\;\mathrm{mod}\;n^{2}$.
- Decryption: Input a cipher text $c\in Z_{n^{2}}^{*}$. Output the plain text $m=Dec\left(c\right)=L\left(c^{\lambda }\;\mathrm{mod}\;n^{2}\right)\cdot \mu \;\mathrm{mod}\;n$.

## 4. Proposed Framework

This section introduces our proposed secure hierarchical asynchronous federated learning (SHAFL) scheme, including design goals, the SHAFL framework, the shuffle model, and the committee consensus mechanism. Table 1 outlines the notation definitions in this study.

### 4.1. Design Goals

The design objectives of SHAFL are as follows:

- Prevent malicious training nodes, gateways, and committee nodes from compromising the local data privacy of training nodes.
- Solve the problem of n−1 collusion attacks among training nodes.
- Eliminate the impact of noise on global model performance.
- Prevent malicious training and committee nodes from compromising system robustness and global model performance.

### 4.2. Framework

**Figure 5:**
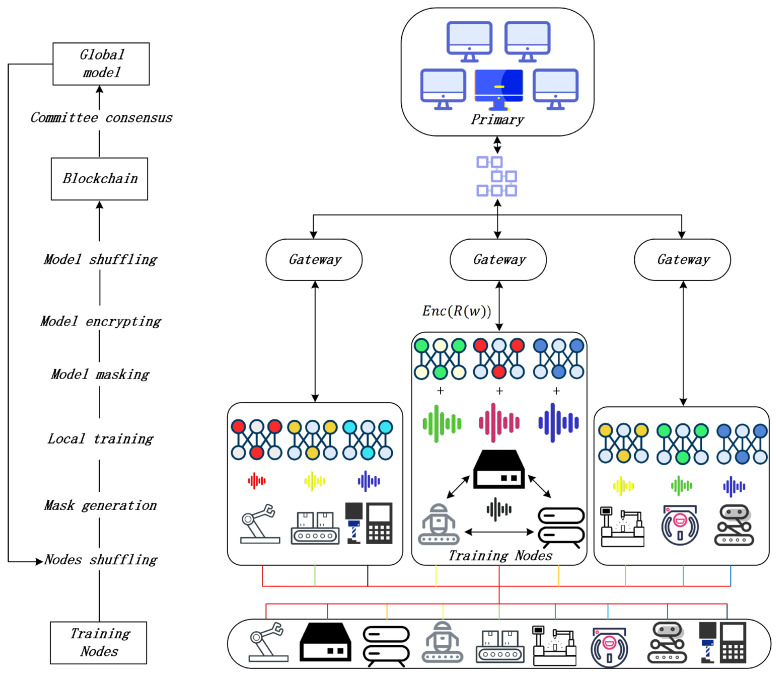
The workflow of iterative SHAFL framework.

The workflow of the SHAFL framework is shown in Figure 5. The SHAFL framework comprises four types of entities: task publishers, committee nodes *U*, training nodes *C*, and gateways *Y*. The task publisher initializes the global model $w^{0}$ (and rewards) in $block_{0}$. The committee nodes share the same Paillier homomorphic key pair upk/usk and act as aggregators. They receive messages from gateways, analyze and aggregate them, and then publish the global model $w^{t}$ and the hyperparameters. Gateways act as shufflers that receive *m* multi-masks from training nodes and upload the output $Z/M_{y_{o}}$ of the shuffle model to the Blockchain. Each training node has the same Paillier homomorphic key pair cpk/csk. The training nodes under the same gateway are called a group $G_{o}$, and the size of the group $G_{o}$ is $s_{o}$. The SHAFL framework is presented in Algorithm 1 with six steps:
**Algorithm 1** Algorithm of SHAFL**Input:** $w^{t-1},H,\left\{D_{c_{i}^{o}}\right\},\epsilon_{c_{i}^{o}},\delta_{c_{i}^{o}},T,O,Y,C,U$ **Output:** $w^{T}$ 1:Task publisher initializes the global model $w^{0}$ (and rewards) in $block_{0}$2:**for** t≤T **do**3:   **for** each $c_{i}\in C,y_{o}\in Y$ **do**4:     $G_{o}=\mathrm{Nodes}\mathrm{shuffling}\left(c_{i},y_{o}\right)$5:   **end for**6:   $u_{q}$ sends the signed messages to Blockchain7:   **for** each $c_{i}^{o}\in G_{o}$ **do**8:     According to $\epsilon_{c_{i}^{o}},\delta_{c_{i}^{o}}$ and Gaussian Mechanism, $c_{i}^{o}$ calculates noise scale $\sigma_{c_{i}^{o}}$9:     $\pi_{c_{i}}^{t-\tau^{o}}$ = **Mask generating**$N(0,\sigma_{c_{i}^{o}}^{2})$10:     $c_{i}^{o}$ receives $\left\{\pi_{c_{j}}^{t-\tau^{o}}\right\}$ from other trainers11:     $c_{i}^{o}$ downloads and decrypts signed messages from Blockchain12:     $w_{c_{i}^{o}}^{t-\tau^{o}}$ = **Local training**($w^{t-1},D_{c_{i}^{o}},H,\gamma$)13:     $\left\{M_{i,k}^{t-\tau^{o}}\right\}$ = **Model masking**($w_{c_{i}^{o}}^{t-\tau^{o}}$,$\pi_{c_{i}}^{t-\tau^{o}}$,$\left\{\pi_{c_{j}}^{t-\tau^{o}}\right\}$)14:     $c_{i}^{o}$ divides and encrypts $\left\{M_{i,k}^{t-\tau^{o}}\right\}$ to $Enc_{upk}\left(\left\{x_{i,k}^{t-\tau^{o},z}\right\}\right)$15:   **end for**16:   **for** each $y_{o}\in G_{o}\subset O$ **do**17:     $w_{y_{o}}^{t-\tau^{o}}=\mathrm{Model}\mathrm{shuffling}\left(Enc_{upk}\left(\left\{x_{i,k}^{t-\tau^{o},z}\right\}\right)\right)$18:     $y_{0}$ signs and saves $w_{y_{o}}^{t-\tau^{o}}$ in Blockchain.19:   **end for**20:   **for** each $u_{i}\in U$ **do**21:     Select $u_{q}$ by $q=hash(block_{t-1})\;\mathrm{mod}\;M$22:     $w^{t},\left\{R_{t,o}^{u_{q}}\right\}$ = **Committee consensus**($U,w^{t-1}$,$\left\{w_{y_{o}}^{t-\tau^{o}}\right\}$)23:   **end for**24:   $u_{q}$ signs and saves $w^{t},\left\{R_{t,o}^{u_{q}}\right\}$ in $block_{t}$25:**end for**26:t=t+127:**return** Outputs

- Node shuffling: In turn *t*, each training node $c_{i}\in C$ randomly selects a gateway as its shuffler. Training nodes under the same gateway $y_{o}$ form a group $G_{o}\subset O$.
- Mask generating: Training nodes process mask–DP exchange protocol. According to the differential privacy parameter $\epsilon_{c_{i}^{o}},\delta_{c_{i}^{o}}$ and the Gaussian mechanism, $c_{i}^{o}$ calculates noise scale $\sigma_{c_{i}^{o}}$ based on Equation (8) and generates masks $\pi_{c_{i}}^{t-\tau^{o}}$ based on Gaussian distribution $N(0,\sigma_{c_{i}^{o}}^{2})$. Then, $c_{i}^{o}$ exchanges masks $\pi_{c_{i}}^{t-\tau^{o}}$ with other training nodes within a group $G_{o}$.
- Local training: All training nodes $\left\{c_{i}^{o}\right\}$ receive the signed and encrypted message from the gateway, decrypt $Dec_{csk}\left(Enc_{cpk}\left(w^{t-1},\gamma ,\tau_{max},H\right)\right)$ with private key csk, obtain the global model $w^{t-1}$, set their learning rate γ, and train the global model $w^{t-1}$ with $D_{c_{i}^{o}}$ locally using Equations (3) and (4).
- Model masking: Training node $c_{i}^{o}$ subsamples its local update $\left\{w_{c_{i}^{o}}^{t-\tau^{o}}\right\}$ and performs z−th dummy layer $\left\{p_{i,0}^{t-\tau^{o},z}\right\}$ filling on the sampled model to restore the original model shape. Using the filled model $w_{c_{i}^{o}}^{*,t-\tau^{o}}$, masks $\pi_{c_{i}}^{t-\tau^{o}}$ and $\left\{\pi_{c_{j}}^{t-\tau^{o}}\right\}$, and the training node generates *m* multi-masks messages according to Equation (10). *m* multi-masks $\left\{M_{i,k}^{t-\tau^{o}}\right\}$ are further divided into *d*-layer vectors $\left\{x_{i,k}^{t-\tau^{o},z}\right\},z\in \left[1,d\right]$, according to the shape of the global model. Then, training node $c_{i}^{o}$ encrypts these messages with the primary committee node’s public key upk and sends the encrypted messages $Enc_{upk}\left\{x_{i,k}^{t-\tau^{o},z}\right\}$ to the gateway $y_{o}$. The subsample, dummy-layer filling, and model masking are proposed in Algorithm 2. It is worth noting that the masks are additive Gaussian noises; the encrypted model has the same shape and location information as the global model.
- Model shuffling: After the gateway receives all messages $Enc_{upk}\left\{x_{i,k}^{t-\tau^{o},z}\right\}$ from the training nodes, the gateway shuffles encrypted messages $Enc_{upk}\left(x_{i,k}^{t-\tau^{o},z}\right)$ using Equation (9), and retains the location information of the layer. Then, the gateway generates a new model $w_{y_{o}}^{t-\tau^{o}}$ and sends it to the Blockchain with a delay $\tau^{o}$ asynchronously. If $\tau^{o}>\tau_{max}$, stale models are discarded.
- Committee consensus: Committee nodes *U* select a primary node $u_{q}$. Primary committee node $u_{q}$ downloads $\eta_{t}$ local updates $\left\{w_{y_{o}}^{t-\tau^{o}}\right\}$ from the Blockchain and decrypts them to obtain $\left\{w_{y_{o}}^{t-\tau^{o},*}\right\}$. Then, $u_{q}$ scores the model $\left\{w_{y_{o}}^{t-\tau^{o},*}\right\}$ and signs and broadcasts the scores $\left\{R_{t,o}^{u_{q}}\right\}$ to other committee nodes. Other committee nodes then re-score the models and reach a consensus on scores $\left\{R_{t,o}^{u_{q}}\right\}$. Once a consensus is reached, primary $u_{q}$ aggregates the local updates $\left\{w_{y_{o}}^{t-\tau^{o},*}\right\}$ as
  (12) $$w^{t}=\left(1-\alpha \right)w^{t-1}+\alpha \frac{\sum_{o=1}^{\eta_{t}}R_{t,o}^{u_{i}}w_{y_{o}}^{t-\tau^{o},*}}{\sum_{o=1}^{\eta_{t}}R_{t,o}^{u_{i}}}$$
  where α denotes the hyperparameter of secure aggregation, and $\eta_{t}$ is the number of local updates uploaded by gateways in turn *t*. Primary committee node $u_{q}$ encrypts and uploads the new global model $Enc_{cpk}\left(w^{t}\right)$ to the Blockchain for the next turn t+1.

**Algorithm 2** Model masking**Input:** $w_{c_{i}^{o}}^{t-\tau^{o}},\pi_{c_{i}}^{t},\left\{\pi_{c_{j}}^{t}\right\},\epsilon ,\varphi$ **Output:** $Enc_{upk}\left(x_{i,k}^{t-\tau^{o},z}\right)$1:**for** each $G_{o}\subset O$ **do**2:   **for** each $c_{i}^{o}\in G_{o}$ **do**3:     Calculated mask $\pi_{i}^{t-\tau^{o}}=\sum_{c_{j}\in G_{o}}\pi_{c_{j},i}^{t-\tau^{o}}$4:     **for** z≤d **do**5:        **if** $z\;\mathrm{mod}\;\left.\lceil \frac{d}{s_{o}}\right\rceil =i$ **then**6:$\;\mathrm{Dropped}x_{i,0}^{t-\tau^{o},z}$ and evaluated σ by Equation (8)7:$\;\mathrm{Generated}\mathrm{dummy}\mathrm{layers}p_{i,0}^{t-\tau^{o},z}=N\left(0,\sigma_{c_{i}}^{2}\right)$8:$\;x_{i,0}^{t-\tau^{o},z}\leftarrow p_{i,0}^{t-\tau^{o},z}$9:endif10:z=z+111:endfor12:$\;\mathrm{Generated}\mathrm{masks}\left\{M_{i,k}^{t-\tau^{o}}\right\}\mathrm{with}w_{c_{i}^{o}}^{*,t-\tau^{o}}$ by Equation (10)13:$\;\mathrm{Divide}\mathrm{masks}\mathrm{as}\left\{x_{i,k}^{t-\tau^{o},z}\right\}\leftarrow \left\{M_{i,k}^{t-\tau^{o},z}\right\}$14:forz≤ddo15:$\;x_{i,k}^{t-\tau^{o},z}\leftarrow \left\lfloor \varphi \cdot x_{i,k}^{t-\tau^{o},z}\right\rfloor$16:endfor17:$\;\mathrm{Encrypted}\{x_{i,k}^{t-\tau^{o},z}\}\mathrm{to}Enc_{upk}\left(x_{i,k}^{t-\tau^{o},z}\right)$ using primary committee’s public key *upk*18:endfor19:$\;\forall c_{i}^{o}\in G_{o}\mathrm{send}\mathrm{Encrypted}\mathrm{messages}Enc_{upk}\left(x_{i,k}^{t-\tau^{0},z}\right)$ to gateway *y_o_* synchronous20:endfor21:**return** Outputs

Once *t* reaches the set parameter *T* or the global model $w^{t}$ converges, the FL ends.

### 4.3. Multi-Shuffle with Subsample and Dummy Layers

To enhance the LDP, the SHAFL framework introduces the shuffle model, which fills the subsample and dummy layers. These privacy-enhancing mechanisms reduce the required noise level in local updates while ensuring the performance preservation of models uploaded by training nodes [67]. However, the subsample will cause missing model layers, which makes it difficult for the committee node to combine and aggregate the local updates into an available model [24]. The SHAFL framework introduces dummy layers to ensure a valid model.

**Figure 6:**
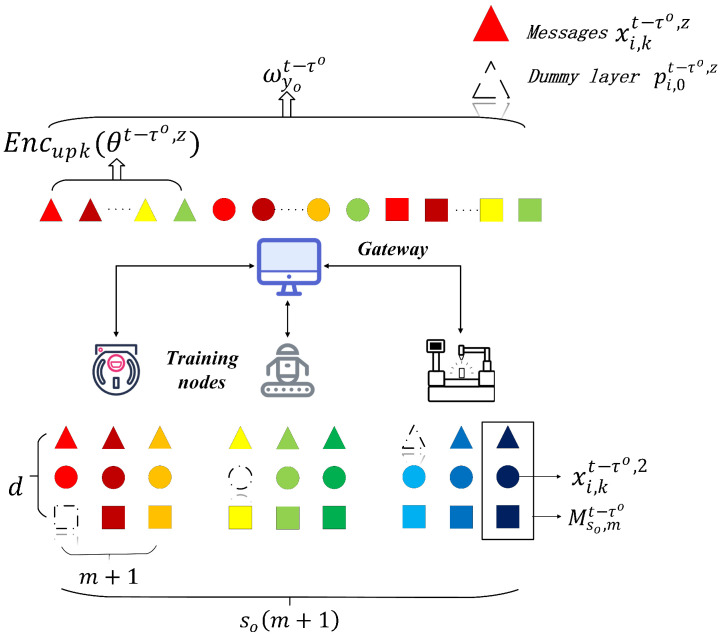
The subsample and dummy-layer filling of the SHAFL framework.

The subsample, dummy-layer filling, and model masking are shown in Figure 6 and Algorithm 2. After local training, the training node first performs continuous layer subsampling on its local updates $w_{c_{i}^{o}}^{t-\tau^{o}}$. All training nodes perform subsampling and drop some model layers. They then evaluate the variance σ of the Gaussian noise using Equation (8), and fill the dropped layers $x_{i,0}^{t-\tau^{o},z}$ with dummy layers $p_{i,0}^{t-\tau^{o},z}$ generated from Gaussian noise $p_{i,0}^{t-\tau^{o},z}=N\left(0,\sigma \right)$, where

(13) $$x_{i,0}^{t-\tau^{o},z}\leftarrow p_{i,0}^{t-\tau^{o},z},\;z=i+r\left\lceil \frac{d}{s_{o}}\right\rceil ,r=0,1,2,\ldots$$

The filled model is denoted as $w_{c_{i}^{o}}^{*,t-\tau^{o}}$. According to the mask–DP exchange protocol, the training node generates *m* multi-masks $\left\{M_{i,k}^{t-\tau^{o}}\right\}$ using masks $\pi_{c_{i}}^{t-\tau^{o}}$ and {$\pi_{c_{j}}^{t-\tau^{o}}$}. It is worth noting that k∈[0,m], and each training node generates m+1 multi-masks {$M_{i,k}^{t-\tau^{o}}$}.

Before uploading the masks $\left\{M_{i,k}^{t-\tau^{o}}\right\}$ to the gateway, the training node divides them layer by layer:

(14) $$x_{i,k}^{t-\tau^{o},z}=M_{i,k}^{t-\tau^{o},z}$$

where $x_{i,k}^{t-\tau^{o},z}$ denotes the z−th layer vector of $M_{i,k}^{t-\tau^{o},z}$, $i\in [1,s_{o}]$, and k∈[0,m], z∈[1,d]. The value of $x_{i,k}^{t-\tau^{o},z}$ is a float number, which can not be encrypted directly with PHE. Therefore, the value of layer vector $x_{i,k}^{t-\tau^{o},z}$ should be expanded to an integer through quantization $x_{i,k}^{t-\tau^{o},z}\leftarrow \left\lfloor \varphi \cdot x_{i,k}^{t-\tau^{o},z}\right\rfloor$, where φ denotes the scale of quantization. Then, using primary committee node $u_{q}$’s homomorphic public key upk, the training node encrypts the layer vector $x_{i,k}^{t-\tau^{o},z}$ to $Enc_{upk}\left\{x_{i,k}^{t-\tau^{o},z}\right\}$ layer by layer and uploads $ds_{o}\left(m+1\right)$ messages to gateway $y_{o}$.

The gateway (shuffler) receives messages from training nodes and shuffles them by layer. Specifically, the gateway will collectively shuffle the order of the layer vectors $Enc_{upk}\left\{x_{i,k}^{t-\tau^{o},z}\right\}$ from all clients at the same layer. After shuffling, according to the hierarchical relationship, the encrypted layer vectors are stored in order to form a new local update $w_{y_{o}}^{t-\tau^{o}}$. Then, the gateway uploads it to the Blockchain. The local update $w_{y_{o}}^{t-\tau^{o}}$ before aggregating is denoted as

(15) $$\;\begin{array}{cc} M_{y_{o}} & =w_{y_{o}}^{t-\tau^{o}}\\ & =A\circ S\circ R^{ds_{o}\left(m+1\right)}\left(X\right)\\ & =A(S_{y_{o}}\left(R_{1}^{d(m+1)}\left(X\right),\ldots ,R_{s_{o}}^{d(m+1)}\left(X\right)\right))\\ & =A(S_{y_{o}}(R_{1}^{d(m+1)}(Enc_{upk}(M_{1,0}^{t-\tau^{o}},M_{1,1}^{t-\tau^{o}},\ldots ,\;\\ & \;\;\;\;M_{1,m}^{t-\tau^{o}})),\ldots ,R_{s_{o}}^{d(m+1)}(Enc_{upk}(M_{s_{o},0}^{t-\tau^{o}},\ldots ,\;\\ & \;\;\;\;M_{s_{o},m}^{t-\tau^{o}}))))\\ & =A(S_{y_{o}}(Enc_{upk}(x_{1,0}^{t-\tau^{o},1},\ldots ,x_{1,0}^{t-\tau^{o},d},\ldots ,x_{1,m}^{t-\tau^{o},1},\;\\ & \;\;\;\;\ldots ,x_{1,m}^{t-\tau^{o},d},\ldots ,x_{s_{o},0}^{t-\tau^{o},1},\ldots ,x_{s_{o},0}^{t-\tau^{o},d},\ldots ,x_{s_{o},m}^{t-\tau^{o},1},\;\\ & \;\;\;\;\ldots ,x_{s_{o},m}^{t-\tau^{o},d})))\\ & =Z \end{array}$$

where *m* is the number of multi-masks, and $x_{s_{o},m}^{t-\tau^{o},z}$ is the z−th layer vector of mask $M_{s_{o},m}^{t-\tau^{o}}$ in turn *t* before encryption. The number of training node messages is d(m+1). The z−th layer of the new local update $w_{y_{o}}^{t-\tau^{o}}$ is

(16) $$\theta^{t-\tau^{o},z}=\prod_{i=1}^{s_{o}}\prod_{k=0}^{m}Enc_{upk}\left(x_{i,k}^{t-\tau^{o},z}\right)$$

Due to homomorphism, $Dec\left(\theta^{t-\tau^{o},z}\right)=\sum_{i=1}^{s_{o}}\sum_{k=0}^{m}\left(x_{i,k}^{t-\tau^{o},z}\right)$

The SHAFL framework uses the gateways as shufflers. The shuffling and pre-aggregation of model $w_{y_{o}}^{t-\tau^{o}}$ are shown in Figure 6 and Algorithm 3.
**Algorithm 3** Model shuffling**Input:** $Y,Enc_{upk}\left(x_{i,k}^{t-\tau^{o},z}\right)$ **Output:** $w_{y_{o}}^{t-\tau^{o}}$1:**for** each $y_{o}\subset Y$ **do**2:   Receives the encrypted messages $\left\{Enc_{upk}\left(x_{i,k}^{t-\tau^{o},z}\right)\right\}$3:   **for** z∈[0,d] **do**4:     Shuffles $\left\{Enc_{upk}\left(x_{i,k}^{t-\tau^{o},z}\right)\right\}$ by Equation (9)5:     $\theta^{t-\tau^{o},z}=\prod_{i=1}^{s_{o}}\prod_{k=0}^{m}Enc_{upk}\left(x_{i,k}^{t-\tau^{o},z}\right)$6:     $w_{y_{o}}^{t-\tau^{o},z}\leftarrow \theta^{t-\tau^{o},z}$7:   **end for**8:   $y_{o}$ uploads local update $w_{y_{o}}^{t-\tau^{o}}$ to Blockchain asynchronous9:**end for**10:**return** Outputs

### 4.4. Committee Consensus

The committee consensus is shown in Algorithm 4. In the t−th round of the SHAFL framework, the primary committee node $u_{q}$ first downloads an $\eta_{t}$ encrypted local update $\left\{w_{y_{o}}^{t-\tau^{o}}\right\}$ from the Blockchain and decrypts it using a homomorphic private key usk layer by layer. Since the layer vectors $\left\{x_{i,k}^{t-\tau^{o},z}\right\}$ are quantized during encryption, mapping floating-point numbers to integers, it is necessary to dequantize the decrypted layer vectors $\left\{\theta^{t-\tau^{o},*,z}\right\}$ to restore them to their original floating-point format. According to the hierarchical relationship, the layer vectors $\left\{\theta^{t-\tau^{o},*,z}\right\}$ are reconstructed into a decrypted local update $w_{y_{o}}^{t-\tau^{o},*}$. Then, the primary committee node $u_{q}$ tests each local update $w_{y_{o}}^{t-\tau^{o},*}$ and global model $w^{t-1}$ using the committee nodes’ local dataset $D_{test}$ to obtain the accuracy $A_{L}$ and $A_{G}$. By using $A_{L}$, $A_{G}$, and the delay $\tau^{o}$, $u_{q}$ calculates the score $R_{t,o}^{u_{q}}$ for each local update $w_{y_{o}}^{t-\tau^{o},*}$ as

(17) $$R_{t,o}^{u_{q}}=\left(\frac{A_{L}}{{\left(A_{L}+A_{G}\right)}^{\tau }}\right)$$

After scoring, the primary committee node $u_{q}$ sends the scores of local updates $\left\{R_{t,o}^{u_{q}}\right\}$ to other committee nodes $\left\{u_{i}\right\}$. $\left\{u_{i}\right\}$ downloads $w_{y_{o}}^{t-\tau^{o},*}$ and re-scores them, and uses a consensus mechanism, PBFT, to reach a consensus on scores $\left\{R_{t,o}^{u_{q}}\right\}$. Once a consensus is reached, $u_{q}$ aggregates the local updates $\left\{w_{y_{o}}^{t-\tau^{o},*}\right\}$ to obtain a new global model $w^{t}$ through secure aggregation using (12). Then, $u_{q}$ encrypts and uploads $Enc_{cpk}\left(w^{t}\right)$ to $block_{t}$.
**Algorithm 4** Committee consensus**Input:** $U,\left\{w_{y_{o}}^{t-\tau^{o}}\right\},w^{t-1},D_{test},s_{o},\varphi$ **Output:** $w^{t},\left\{R_{t,o}^{u_{q}}\right\}$ 1:Primary committee node $u_{q}$ downloads $\eta_{t}$ local updates $\left\{w_{y_{o}}^{t-\tau^{o}}\right\}$ from Blockchain and decrypts it2:**for** $o\leq \eta_{t}$ **do**3:   **for** z≤d **do**4:     $\theta^{t-\tau^{o},*,z}=Dec_{usk}\left(\theta^{t-\tau^{o},z}\right)=\;\sum_{i=1}^{s_{o}}\sum_{k=0}^{m}\left(x_{i,k}^{t-\tau^{o},z}\right)$5:     Dequantization:6:     $\theta^{t-\tau^{o},*,z}=\frac{\theta^{t-\tau^{o},*,z,}}{s_{o}\cdot \varphi }$7:     $w_{y_{o}}^{t-\tau^{o},*,z}\leftarrow \theta^{t-\tau^{o},*,z}$8:     z=z+19:   **end for**10:   Obtain the decrypted local update $w_{y_{o}}^{t-\tau^{o},*}$ of gateway $y_{o}$11:   o=o+112:**end for**13:   Tests the accuracy of global model $w^{t-1}$ by $D_{test}$ to obtain $A_{G}$14:   **for** $o\leq \eta_{t}$ **do**15:     $u_{q}$ test the accuracy of $w_{y_{o}}^{t-\tau^{o},*}$ by $D_{test}$ to obtain $A_{L}$16:     Calculates score $R_{t,o}^{u_{q}}$ of model $w_{y_{o}}^{t-\tau^{o},*}$ by Equation (17)17:     o=o+118:   **end for**19:   Sent scores to all committee node $u_{i}$20:   **for** $u_{i}\in U$ **do**21:     Re-score each local update $w_{y_{o}}^{t-\tau^{o},*}$ by $D_{t}est$22:     Sent scores $\left\{R_{t,o}^{u_{i}}\right\}$ to other committee node23:   **end for**24:   All $u_{i}\in U$ reach a consensus on scores $\left\{R_{t,o}^{u_{q}}\right\}$25:   Pirmary node $u_{q}$ process secure aggregation by Equation (12)26:   $u_{q}$ encrypts the global model to $Enc_{cpk}\left(w^{t}\right)$ by training nodes’ public key27:   Uploads $Enc_{cpk}\left(w^{t}\right)$ to $block_{t}$ 28:**return** Outputs

## 5. Convergence Analysis

In this section, we present the theorem and proof for the convergence analysis of the SHAFL framework.

**Definition 4**  (*L*-smooth [1])**.** *Function f is L-smooth if $\forall x,y\in R^{N},x\neq y$ exists:*

(18) $$f\left(y\right)\leq f\left(x\right)+\left(y-x\right)\nabla f\left(x\right)+\frac{L}{2}{\left\|y-x\right\|}^{2}$$

**Definition 5**  (μ-strongly convex [1]). *Function f is μ-strongly convex if $\forall x,y\in R^{N},x\neq y$ exists:*

(19) $$f\left(y\right)\geq f\left(x\right)+\left(y-x\right)\nabla f\left(x\right)+\frac{\mu }{2}{\left\|y-x\right\|}^{2}$$

**Theorem 1.**  *Assume the global loss function F is L-smooth and μ-strongly convex. For the group $G_{o}$, let the learning rate be $\gamma <\frac{1}{L}$ and the local iterations be $H\in [H_{min},H_{max}]$. For $\forall w\in R^{d},\forall \xi \in D$, the expected square norm of the gradients is bounded:*

(20) $$E_{\xi \in D}{\left\|\nabla f\left(w,\xi \right)\right\|}^{2}\leq Q$$

For the initial global model $w^{0}$ and optimization model $w^{*}$

(21) $$F\left(w^{0}\right)-F\left(w^{*}\right)\geq {\left(1-\alpha \right)}^{T}\frac{\gamma }{2}HQ$$

*After T turns, the convergence bond of the global loss function is*

(22) $$\begin{array}{cc} E & \left[F\left(w^{T}\right)-F\left(w^{*}\right)\right]\\ & \leq F\left(w^{0}\right)-F\left(w^{*}\right)+\left[1-{\left(1-\alpha \right)}^{T}\right]\frac{\gamma }{2}HQ \end{array}$$

**Proof of Theorem 1.**  Since prior studies [1,70] have established convergence analysis for hierarchical asynchronous federated learning frameworks, we specifically focus on presenting several distinct components in this study. For a training node $c_{i}^{o}$ in an arbitrary group $G_{o}$, after performing *H* a local update, the convergence bound is

(23) $$\begin{array}{cc} E & \left[F\left(w_{c_{i}^{o}}^{t-\tau^{o},H}\right)-F\left(w^{*}\right)\right]\\ & \leq F\left(w_{c_{i}^{o}}^{t-\tau^{o},0}\right)-F\left(w^{*}\right)-\frac{\gamma }{2}HQ \end{array}$$

where $w_{c_{i}^{o}}^{t-\tau^{o},H}$ is derived from $w_{c_{i}^{o}}^{t-\tau^{o},0}$ by *H* iterations. Then, the committee nodes will aggregate $\eta_{t}$ local updates $\left\{w_{y_{o}}^{t-\tau^{o}}\right\}$ from the gateway to obtain a new global model $w^{t}$. Thus, the convergence bound of the SHAFL framework after *t* turns is

(24) $$\begin{array}{cc} E & [F\left(w^{t}\right)-F\left(w^{*})\right]\\ & \leq \left(1-\alpha \right)F\left(w^{t-1}\right)+\alpha E\left[F(\frac{\sum_{o=1}^{\eta_{t}}R_{t,o}^{u_{i}}w_{y_{o}}^{t-\tau^{o}}}{\sum_{o=1}^{\eta_{t}}R_{t,o}^{u_{i}}})\right]-F\left(w^{*}\right)\\ & \leq \left(1-\alpha \right)F\left(w^{t-1}\right)+\alpha \sum_{o=1}^{\eta_{t}}\frac{R_{t,o}^{u_{i}}F\left(w_{y_{o}}^{t-\tau^{o}}\right)}{\sum_{o=1}^{\eta_{t}}R_{t,o}^{u_{i}}}-F\left(w^{*}\right)\\ & \leq \left(1-\alpha \right)F\left(w^{t-1}\right)+\alpha E\left[F(w_{y_{o}}^{t-\tau^{o}})\right]-F\left(w^{*}\right)\\ & \leq \left(1-\alpha \right)F\left(w^{t-1}\right)+\alpha E\left[F(\frac{\sum_{i=1}^{s_{o}}\left|D_{c_{i}^{o}}\right|\sum_{k=1}^{m}M_{i,k}^{t-\tau^{o}}}{\sum_{i=1}^{s_{o}}\left|D_{c_{i}^{o}}\right|})\right]-F\left(w^{*}\right)\\ & \leq \left(1-\alpha \right)F\left(w^{t-1}\right)+\alpha E\left[F(\frac{\sum_{i=1}^{s_{o}}\left|D_{c_{i}^{o}}\right|w_{c_{i}^{o}}^{t-\tau^{o}}}{\sum_{i=1}^{s_{o}}\left|D_{c_{i}^{o}}\right|})\right]-F\left(w^{*}\right)\\ & \leq \left(1-\alpha \right)F\left(w^{t-1}\right)+\alpha E\left[F(w_{c_{i}^{o}}^{t-\tau^{o}})\right]-F\left(w^{*}\right)\\ & \leq \left(1-\alpha \right)\left[F\left(w^{t-1}\right)-F\left(w^{*}\right)\right]+\alpha E\left[F\left(w_{c_{i}^{o}}^{t-\tau^{o}}\right)-F\left(w^{*}\right)\right] \end{array}$$

Using Equations (23) and (24), after performing *T* global turns, the convergence bound of the SHAFL framework is

(25) $$\begin{array}{cc} E & [F\left(w^{T}\right)-F\left(w^{*}\right)]\\ & \leq \left(1-\alpha \right)\left[F\left(w^{T-1}\right)-F\left(w^{*}\right)\right]+\alpha E\left[F\left(w_{c_{i}^{o}}^{T-\tau^{o}}\right)-F\left(w^{*}\right)\right]\\ & \leq \left(1-\alpha \right)\left[\left(1-\alpha \right)\left[F\left(w^{T-2}\right)-F\left(w^{*}\right)\right]+\alpha E\left[F\left(w_{c_{i}^{o}}^{T-1-\tau^{o}}\right)-F\left(w^{*}\right)\right]\right]\;\\ & \;\;\;\;+\alpha E[F\left(w_{c_{i}^{o}}^{T-\tau^{o}}\right)-F\left(w^{*}\right)]\\ & \leq \left(1-\alpha \right)\left(1-\alpha \right)\left[F\left(w^{T-2}\right)-F\left(w^{*}\right)\right]+\left(1-\alpha \right)\alpha E\left[F\left(w_{c_{i}^{o}}^{T-1-\tau^{o}}\right)-F\left(w^{*}\right)\right]\;\\ & \;\;\;\;+\alpha E[F\left(w_{c_{i}^{o}}^{T-\tau^{o}}\right)-F\left(w^{*}\right)]\\ & \leq {\left(1-\alpha \right)}^{T}\left[F\left(w^{0}\right)-F\left(w^{*}\right)\right]+\left[\alpha +\left(1-\alpha \right)\alpha +\ldots +\left(T-1\right)\left(1-\alpha \right)\alpha \right]E[F\left(w_{c_{i}^{o}}^{t-\tau^{o},H}\right)\;\\ & \;\;\;\;-F(w^{*})]\\ & \leq {\left(1-\alpha \right)}^{T}\left[F\left(w^{0}\right)-F\left(w^{*}\right)\right]+\left[1-{\left(1-\alpha \right)}^{T}\right]\left[F\left(w_{c_{i}^{o}}^{t-\tau^{o},0}\right)-F\left(w^{*}\right)-\frac{\gamma }{2}HQ\right]\\ & \leq {\left(1-\alpha \right)}^{T}\left[F\left(w^{0}\right)-F\left(w^{*}\right)\right]+\left[1-{\left(1-\alpha \right)}^{T}\right]\left[F\left(w^{0}\right)-F\left(w^{*}\right)-\frac{\gamma }{2}HQ\right]\\ & \leq F\left(w^{0}\right)-F\left(w^{*}\right)+\left[1-{\left(1-\alpha \right)}^{T}\right]\frac{\gamma }{2}HQ] \end{array}$$

Thus, Theorem 1 derives the convergence bound after *T* turns. □

## 6. Security Analysis

This section describes the security analysis of the SHAFL framework as follows: privacy-preserving analysis, system robustness analysis, and model security analysis.

### 6.1. Privacy-Preserving Analysis

**Lemma 1**  (Amplification by shuffling [58])**.** *Let R be an $\epsilon_{0}$-LDP mechanism. Then, the shuffle model $M\left(x_{1},\ldots ,x_{n}\right):=A\circ S\circ \left(R\left(x_{1}\right),\ldots ,R\left(x_{n}\right)\right)$ satisfies (ϵ,δ)-DP, where*

- *If*
(26) $$\epsilon_{0}\leq \frac{\log(n/log(2/\delta ))}{2}$$
*for any δ>0, it has*
(27) $$\epsilon =O(\min\left\{\epsilon_{0},1\right\}e^{\epsilon_{0}}\sqrt{\frac{log(1/\delta )}{n}}).$$

**Lemma 2**  (Amplification by subsampling [60])**.** *If $M:X^{n}\to Y$ satisfies (ϵ,δ)-DP with the relationship on the set n, then $M^{\prime }:X^{m}\to Y$ satisfies $\left(\log\left(1+\left(n/m\right)\left(e^{\epsilon }-1\right)\right),\left(n/m\right)\delta \right)$-DP.*

**Theorem 2.**  *In the SHAFL framework, the training node employs the Gaussian mechanism-based $\left(\epsilon_{l},\delta_{l}\right)$-LDP to preserve data privacy. Through the integration of the shuffle model and subsample, the privacy parameters of the local model satisfy*

(28) $$\begin{array}{cc} \epsilon_{c}= & O(min\left\{\log(1+\left(\left\lceil \frac{d}{s_{o}}\right\rceil -1\right)\left(e^{\epsilon_{l}}-1\right)),1\right\}[1+\left(\left\lceil \frac{d}{s_{o}}\right\rceil -1\right)(e^{\epsilon_{l}}\;\\ & -1)]\sqrt{\frac{\log(1/\left(\left\lceil \frac{d}{s_{o}}\right\rceil -1\right)\delta_{l})}{(m+1)d}}) \end{array}$$

(29) $$\delta_{c}=\left(\left\lceil \frac{d}{s_{o}}\right\rceil -1\right)\delta_{l}$$

Equations (28) and (29) demonstrate the conversion relationship between local privacy parameters $\left(\epsilon_{l},\delta_{l}\right)$ and central differential privacy parameters $\left(\epsilon_{c},\delta_{c}\right)$.

**Proof of Theorem 2.**  In Algorithm 2, $d/\left\lceil \frac{d}{s_{o}}\right\rceil$ layers of the model are dropped and replaced with dummy layers $\left\{p_{i,0}^{t-\tau^{o},z}\right\}$. Therefore, the SHAFL framework samples $d-d/\left\lceil \frac{d}{s_{o}}\right\rceil$ layers from the model parameter space. According to Lemma 2, the local model satisfies

(30) $$\begin{array}{cc} \epsilon_{sub} & =\log(1+\frac{d-d/\left\lceil \frac{d}{s_{o}}\right\rceil }{d}\left(e^{\epsilon_{l}}-1\right))\;\\ & =\log(1+\left(\left\lceil \frac{d}{s_{o}}\right\rceil -1\right)\left(e^{\epsilon_{l}}-1\right)) \end{array}$$

(31) $$\begin{array}{cc} \delta_{sub} & =\frac{d-d/\left\lceil \frac{d}{s_{o}}\right\rceil }{d}\delta_{l}\;\\ & =\left(\left\lceil \frac{d}{s_{o}}\right\rceil -1\right)\delta_{l} \end{array}$$

where $\frac{d-d/\left\lceil \frac{d}{s_{o}}\right\rceil }{d}$ is the subsampling rate. After subsampling, the local model satisfies $\left(\epsilon_{sub},\delta_{sub}\right)$-DP. Since the training nodes send the subsampled model to the gateway, the gateway performs a random permutation on the subsampled model. According to Lemma 1, the local model processed with subsampling and shuffling satisfies

(32) $$\begin{array}{cc} \epsilon_{c} & =O(min\left\{\epsilon_{sub},1\right\}e^{\epsilon_{sub}}\sqrt{\frac{\log(1/\delta_{sub})}{(m+1)d}})\\ & =O(min\left\{\epsilon_{sub},1\right\}e^{\epsilon_{sub}}\sqrt{\frac{\log(1/\left(\left\lceil \frac{d}{s_{o}}\right\rceil -1\right)\delta_{l})}{(m+1)d}})\\ & =O(min\left\{\log(1+\left(\left\lceil \frac{d}{s_{o}}\right\rceil -1\right)\left(e^{\epsilon_{l}}-1\right)),1\right\}[1+\left(\left\lceil \frac{d}{s_{o}}\right\rceil -1\right)(e^{\epsilon_{l}}\;\\ & -1)]\sqrt{\frac{\log(1/\left(\left\lceil \frac{d}{s_{o}}\right\rceil -1\right)\delta_{l})}{(m+1)d}}) \end{array}$$

(33) $$\begin{array}{cc} \delta_{c} & =\delta_{sub}\\ & =\left(\left\lceil \frac{d}{s_{o}}\right\rceil -1\right)\delta_{l} \end{array}$$

as shown in Theorem 2. □

### 6.2. System Robustness Analysis

The SHAFL framework introduces a novel secure aggregation algorithm. Before the committee nodes aggregate a new global model $w^{t}$, the primary committee node $u_{q}$ evaluates the test accuracy of each local update $\left\{w_{y_{o}}^{t-\tau^{o}}\right\}$ using a globally shared test dataset $D_{test}$. The algorithm then calculates a score for each model based on its accuracy and delay $\tau^{o}$, which serves as the aggregation weight of $\left\{w_{y_{o}}^{t-\tau^{o}}\right\}$. A suboptimal model uploaded by a malicious training node will achieve low test accuracy and therefore receive a low aggregation weight. The SHAFL framework mitigates the impact of malicious training nodes on the global model’s performance using the secure aggregation algorithm described above. In the latter rounds of training, the accuracy of both the global model and the local models becomes high and similar. The aggregation weight of the model uploaded by $y_{o}$ with a high delay is significantly lower than that of normal models, thereby mitigating the detrimental impact of stale models on the performance of the global model.

In the event of node disconnections after the mask–DP exchange protocol, the mask–DP introduced by the SHAFL framework is equivalent to a Gaussian noise-based DP. When training node $c_{i}^{o}$ is offline, each training node generates noise with a variance of

(34) $$\sigma =\frac{m\sigma_{c_{i}^{o}}}{s_{o}-1}+\frac{\sum_{n=i}^{i+m}\sigma_{c_{n}}}{m+1}$$

where σ is the variance of noises π, and *m* is the number of exchange masks.

### 6.3. Model Security Analysis

The proposed SHAFL framework employs a combination of consortium Blockchain technology, HE, and DP-based masks to ensure the privacy and security of local data for training nodes. The consortium Blockchain, as a private chain, restricts data access to authorized nodes only, thereby mitigating privacy threats from external nodes. ﻿ Within the SHAFL framework, all committee nodes and training nodes each possess homomorphic key pairs <upk,usk>,<cpk,csk>. When committee nodes distribute the global model to training nodes via an intermediate gateway node, they encrypt the global model with the training node’s homomorphic public key cpk. Except for the training node, no one else can access the global model. Before sending local updates to the gateway, all training nodes encrypt their messages using the committee nodes’ homomorphic public key upk, preventing the gateway and the training nodes from extracting any original model information. The gateway shuffles received messages from training nodes and disrupts the mapping between messages and training nodes. The committee nodes can only receive the shuffled model from the gateway, not the original model from the training node. If the gateway and committee nodes collude, they can use the committee node’s private usk key to decrypt the local updates uploaded by the training nodes. However, since the local updates uploaded by the training nodes are masked, they cannot obtain the original local updates of the training nodes.

## 7. Experiments

### 7.1. Experimental Setting

#### 7.1.1. Benchmarks

The baseline algorithms used in the experiments are introduced as follows.

- FedAvg [62], as the canonical synchronous federated learning framework, was adopted as the baseline comparative scheme in our experiments. This implementation deliberately excludes privacy-preserving mechanisms and Byzantine fault tolerance capabilities.
- DP–FedAvg [71] is a privacy-preserving federated learning framework based on LDP. By injecting noise into their local models, training nodes ensure that the uploaded local models satisfy LDP requirements, thereby defending against inference attacks from the server.
- FedSDP [24] is a synchronous privacy-preserving federated learning framework designed for the Internet of Vehicles (IoV), which enhances privacy and improves data utility through a tripartite mechanism that combines Top-k gradient subsampling, virtual point padding, and shuffle-based anonymization.
- MSFL [61] is a privacy-preserving federated learning framework that synergistically integrates multi-stage shuffling mechanisms and Byzantine-resilient consensus algorithms. It enhances privacy by shuffling training nodes and local updates.
- PBFL [27] is a synchronous, centralized privacy-preserving federated learning framework that achieves privacy-preserving through HE and ensures Byzantine fault tolerance via cosine similarity-based gradient validation.
- PPAFL [18] is an asynchronous privacy-preserving federated learning framework that implements LDP via the Laplace mechanism.
- RAFLS [34] is an RDP-based adaptive FL scheme. It uses the sensitivity of different layers’ weights to determine the amount of noise injected into the model, adopts a model-parameter shuffling mechanism to achieve local model anonymity, and proposes a fine-grained model-weight aggregation scheme.

Table 2 compares the computational complexity of the evaluated schemes from three aspects: local training, aggregation, and privacy preserving. The FedSGP scheme’s marginally higher local training loss is a consequence of the additional Tok sparsification operation performed locally. Regarding aggregation and privacy preservation, due to the use of homomorphic encryption, PBFL and SHAFL exhibit significantly higher computational complexity than other schemes.

#### 7.1.2. Datasets and Models

Three benchmark datasets were rigorously employed in our experiments: MNIST [72], CIFAR-10 [73], and a Heart Disease dataset [74]. The MNIST dataset is a classic handwritten digital image dataset, comprising a training set of 60,000 grayscale images and a test set of 10,000 grayscale images, each standardized to a resolution of 28 × 28 pixels. The test set of 10,000 grayscale images is used to form $D_{test}$. The committee nodes utilize $D_{test}$ to evaluate the accuracy of local updates uploaded by the gateways and assign aggregation weights to each gateway’s local updates based on their accuracy. The training set comprising 50,000 images is evenly distributed across the training nodes. The training nodes then conduct training using their allocated subsets of the training data. The model used on the MNIST dataset is a two-layer CNN. The CIFAR-10 dataset includes 60,000 labeled RGB images (32 × 32 pixels) across 10 object classes, which are divided into 50,000 training images and 10,000 test images. For the CIFAR-10 dataset, the partitioning method for $D_{test}$ and the training dataset is the same as that for the MNIST dataset. The model architecture employed on the CIFAR-10 dataset is ResNet-18. The Heart Disease dataset is a real-world IoMT dataset. The dataset contains approximately 37,000 heart activity samples, each with a 50-dimensional feature vector including heart rate, body mass index, glucose levels, and a label indicating coronary heart disease. There are 1,500 heart health samples across these sample nodes. For the Heart Disease dataset, the model and dataset partitioning scheme are adopted from Reference [74].

#### 7.1.3. Experimental Parameters

The experiment was implemented with Python 3.9 and PyTorch 2.1.0 on a computer equipped with an Intel CPU i5-12400F (Santa Clara, CA, USA) and a NVIDIA GPU 3060Ti (Santa Clara, CA, USA). The random seed was 42, and the key size was 2048-bit, as referenced in [75]. Different experimental parameters were adopted for the three datasets, as shown in Table 3, Table 4, Table 5, where N denotes the number of training nodes, M denotes the number of committee nodes, H denotes number of local iterations, χ denotes the proportion of malicious nodes within the training node set, λ denotes the aggregation hyperparameter of FedAvg [62], T denotes number of global iterations, γ denotes learning rate, α denotes the aggregation hyperparameter of SHAFL, ϵ,δ denotes differential privacy parameters, and $\tau_{max}$ denotes the maximum aggregation delay.

### 7.2. Experimental Result

#### 7.2.1. Performance Analysis

In the absence of malicious nodes, Table 6 presents the model accuracy of each scheme across three datasets. Except for the non-privacy-preserving baseline scheme FedAvg, all other schemes employ a privacy budget of ϵ=1 and $\delta =1\times 10^{-3}$, coupled with a subsampling rate of 0.9. As evidenced by Table 6, under identical privacy budget conditions, the proposed scheme achieved superior model accuracy across all three datasets compared to other schemes, with the exception of the non-privacy-preserving baseline FedAvg.

**Figure 7:**
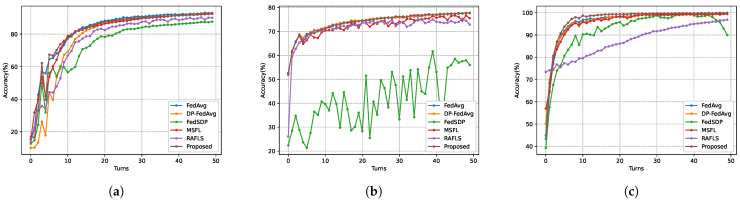
Comparison of the model testing accuracy of each method under three datasets, where ϵ=1, χ=0, (**a**) corresponds to the MNIST dataset, (**b**) corresponds to the CIFAR-10 dataset, and (**c**) corresponds to the Heart Disease dataset.

Figure 7 illustrates the global model performance of five schemes across three datasets. As observed in Figure 7a on the MNIST dataset, the proposed SHAFL framework achieved comparable accuracy to the non-privacy-preserving baseline FedAvg, with a marginal difference of merely 0.26%. Furthermore, after the 40th training iteration, SHAFL, MSFL, and FedAvg all showed convergence in model accuracy. This demonstrates that the SHAFL framework maintains strong model utility and convergence properties under identical privacy budget constraints. Similarly, as depicted in Figure 7b,c, the SHAFL framework demonstrates robust performance on both the CIFAR-10 dataset and the Heart Disease dataset. Notably, on CIFAR-10, the model accuracy of the SHAFL framework surpasses FedAvg by a narrow margin of 0.06%, which can be attributed to the enhanced generalization capability enabled by the minimal noise injection. Additionally, SHAFL, FedAvg, and DP-FedAvg all converged around the 40th training iteration, collectively demonstrating stable optimization trajectories. After the 10th round, both MSFL and RAFLS exhibited persistent oscillations. This occurs because, as the model approaches convergence, excessive noise injection causes the model parameters to fluctuate around the optimum. In contrast, the SHAFL scheme employs eliminable noise, thereby effectively mitigating the occurrence of oscillations. On the Heart Disease dataset, the SHAFL framework exhibited 0.18% lower model accuracy than the non-privacy-preserving baseline FedAvg, yet outperformed all other comparative schemes. Additionally, the SHAFL framework demonstrated a marginally faster convergence rate than the remaining approaches. In conclusion, compared with the baseline approach, FedAvg and the SHAFL framework achieved comparable model accuracy while providing enhanced privacy protection for local data on training nodes. Compared with other privacy-preserving schemes under the same privacy budget, the SHAFL framework achieved higher model accuracy and superior convergence properties.

#### 7.2.2. Impact of Sampling Strategies on Model Accuracy

**Figure 8:**
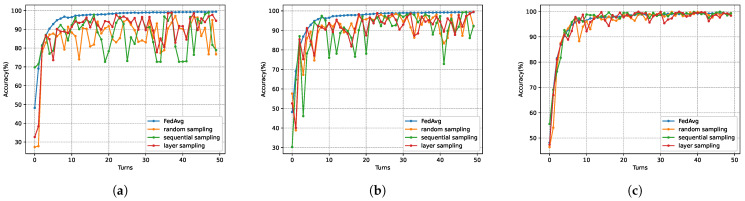
Model accuracy of different sampling strategies on the MNIST dataset, where (**a**) corresponds to ϵ=0.5, (**b**) corresponds to ϵ=0.8, and (**c**) corresponds to ϵ=1.

**Figure 9:**
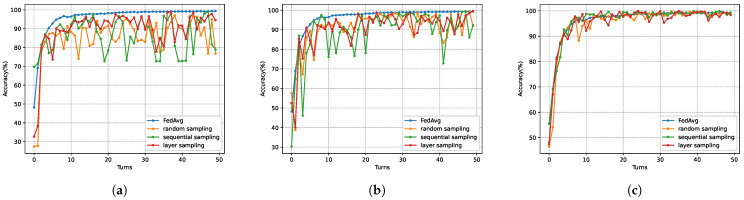
Model accuracy of different sampling strategies on the Heart Disease dataset, where (**a**) corresponds to ϵ=0.5, (**b**) corresponds to ϵ=0.8, and (**c**) corresponds to ϵ=1.

We evaluated the impact of three subsampling strategies on model accuracy. In the experiments, the model fixed the local noise variance and adjusted the scheme’s privacy budget to control the sampling rate. Three privacy budget values, ϵ=0.5,0.8, and 1, were selected, corresponding to sampling rates of 63%,70%, and 90%, respectively. Figure 8 and Figure 9 illustrate the impact of different sampling strategies on model performance across datasets. As shown in Figure 8 and Figure 9, under privacy budgets of ϵ=0.5 and 0.8, the layer sampling proposed by the SHAFL framework achieved a significant improvement in model accuracy compared to other schemes, while exhibiting smaller oscillation amplitudes. At ϵ=1, the accuracy of layer sampling outperforms sequential sampling and matches the baseline FedAvg scheme without sampling.

#### 7.2.3. Analysis of Byzantine Attack Resistance

**Figure 10:**
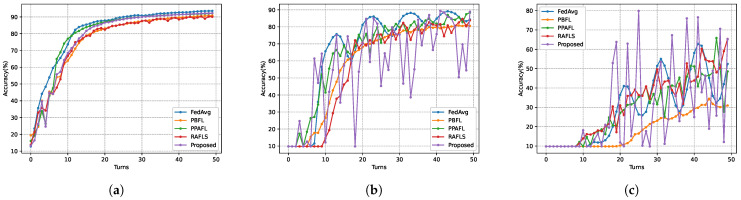
Model accuracy of different χ on MNIST dataset, where (**a**) corresponds to χ=0, (**b**) corresponds to χ=0.2, and (**c**) corresponds to χ=0.4.

**Figure 11:**
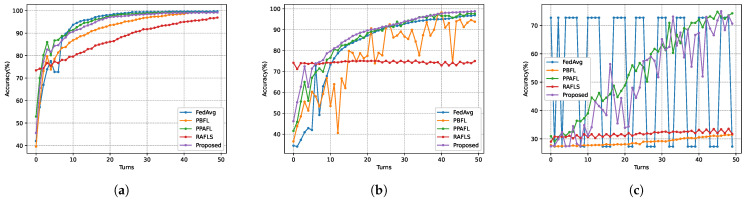
Model accuracy of different χ on Heart Disease dataset, where (**a**) corresponds to χ=0, (**b**) corresponds to χ=0.2, and (**c**) corresponds to χ=0.4.

To evaluate the Byzantine attack resistance of the models, it compared model accuracy across several schemes at varying proportions of malicious nodes. FedAvg served as the baseline to reflect the Byzantine robustness of other schemes in asynchronous environments. In the experiments, it set the maximum number of delay rounds $\tau_{max}=3$ and the privacy budget ϵ=1. As shown in Figure 10 and Figure 11, all schemes experienced a significant drop in model accuracy at Turn 4. For instance, FedAvg in Figure 10 achieved an accuracy of 72.73% at Turn 6, but this plummeted to 49.18% at Turn 7, marking a 48.18% decline. These results indicate that the participation of stale models in aggregation during early training stages degrades accuracy more severely than the impact of a limited number of malicious nodes. From Figure 10 on the MNIST dataset, when χ=0, the accuracy of the SHAFL framework is 1.37% lower than FedAvg but 1.42% higher than PBFL. At χ=0.2, the SHAFL framework outperforms FedAvg, PBFL, and PPAFL by 4.59%, 8.35%, and 0.52%, respectively. When χ=0.4, the accuracy of the SHAFL framework surpasses FedAvg, PBFL, PPAFL and RAFLS by 12.75%, 34.26%, 16.62%, and 5.07%, respectively. Similarly, as shown in Figure 11, when χ=0.2, the accuracy of the SHAFL framework surpasses FedAvg, PBFL, PPAFL and RAFLS by 71.81%, 67.36%, 24.54%, and 67.09%, respectively. Figure 11 demonstrates that the SHAFL framework achieves higher accuracy than other schemes in environments with malicious nodes. Notably, when χ=0.4, the accuracy of the SHAFL framework exceeds FedAvg and PBFL by 43.37% and 39.19%, respectively. This superiority stems from the SHAFL framework’s mechanism: it evaluates each gateway-uploaded model’s accuracy on a test dataset before computing aggregation weights. This approach assigns lower aggregation weights to models from gateways that contain malicious nodes, thereby minimizing their influence on the global model.

#### 7.2.4. Privacy Enhancement Analysis

**Figure 12:**
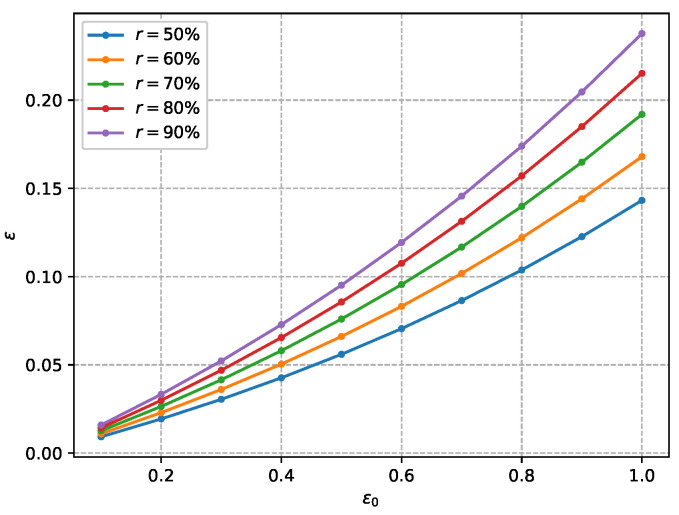
Comparison of privacy enhancement with different subsampling rates.

As demonstrated in Figure 12, the correlation between local and central privacy across varying sampling rates shows that the local privacy budget of training nodes is significantly reduced by the subsampling mechanism and the shuffling model. This substantiates SHAFL’s inherent privacy-enhancing capability. Furthermore, the experimental results show that SHAFL’s privacy amplification effect strengthens as the subsampling rate decreases. This phenomenon occurs because lower sampling rates inherently retain fewer model parameters during aggregation, thereby containing a correspondingly lower amount of sensitive information susceptible to privacy leakage.

## 8. Conclusions

This study proposes a secure asynchronous hierarchical federated learning (SHAFL) framework. In the first layer, it introduces a decentralized mask–DP exchange protocol. Under a gateway, training nodes generate masks using the Gaussian mechanism and exchange them according to the protocol. Each training node then constructs a set of messages using its locally generated mask and those received from other nodes, such that their aggregation recovers the original local model without noise perturbation. To prevent gateways and training nodes from inferring private information from uploaded messages, it employs homomorphic encryption. At the gateway, a shuffling mechanism is applied to disrupt the order of uploaded messages, further enhancing the privacy-preserving level for the local models. In the second layer, it implements an accuracy-based, committee-consensus scoring mechanism, where the primary committee node uses a global test dataset to evaluate and score models uploaded by gateways, thereby determining their aggregation weights. This reduces the impact of malicious nodes on the global model. Theoretical analysis and experimental results demonstrate that our proposed SHAFL achieves superior performance in privacy-preserving and Byzantine-robustness. However, as our scheme employs the Paillier homomorphic encryption algorithm to resist collusion attacks, it incurs relatively high computational overhead. Additionally, our experimental results are obtained using an IID dataset, without considering the impact of Non-IID datasets on the convergence of model aggregation. In future work, we plan to explore ways to reduce computational cost under the existing security assumptions, while also accounting for the effects of non-IID datasets when designing the aggregation scheme. 

## Figures and Tables

**Table 1:** Nomenclature.

Notation	Description	Notation	Description
*F*	global loss	$c_{i}$,$c_{i}^{o}$	i-th trainer; *i*-th trainer in $G_{o}$
ρ	weights	$y_{o}$	gateway of $G_{o}$
*f*	loss function	$G_{o}$	*o*-th group
*w*	model & model parameters	*h*	h-th iteration
$w^{t}$	global model for t-th turn	*t*	t-th turn
$w_{c_{i}^{o}}^{t-\tau^{o},h}$	local update for c in h-th iteration t-th turn	ξ	sample of dataset
$w_{y_{o}}^{t-\tau^{o}}$	local update for group $G_{o}$	$\tau^{o}$	delay of group $G_{o}$
$M_{i,k}^{t-\tau^{o}}$	*m*-multi-masks	$R_{t,o}^{u_{i}}$	score of model $w_{y_{o}}^{t-\tau^{o}}$ generated by $u_{i}$
$x_{i,k}^{t-\tau^{o},z}$	vector of z−th layer of mask $M_{i,k}^{t-\tau^{o}}$	$v_{z}$	number of $x_{i,r}^{t-\tau^{o},z}$
$u_{q}$	primary committee node	$u_{i}$	committee nodes
$D_{test}$	global datasets	$s_{o}$	size of group $G_{o}$
$D_{c_{i}^{o}}$	local dataset for $c_{i}^{o}$	*C*	set of training nodes
α	hyperparameters of aggregation	*U*	set of committees
$\gamma_{c_{i}^{o}}$	learning rate of training node $c_{i}^{o}$	*N*	number of training nodes
$\eta_{t}$	number of $w_{y_{o}}^{t-\tau^{o}}$ collected in *t*-th turn	*M*	number of committee nodes
σ	variance of noise	*m*	number of exchange noises
$\pi_{c_{i}}^{t-\tau^{o}}$	mask generated by $c_{i}^{o}$	*K*	number of groups
*O*	set of groups	*Y*	set of gateways
$\theta^{t-\tau^{o},z}$	z−th layer of model $w_{y_{0}}^{t-\tau^{o}}$	$p_{i,0}^{t-\tau^{o},z}$	z−th dummy layer of model $w_{c_{i}^{o}}^{t-\tau^{o}}$
$H_{min}$	minimum training iteration	$H_{max}$	maximum training iteration

**Table 2:** Computational complexity.

Scheme	Local Train	Aggregation	Privacy Preserving
FedAvg [62]	O(|w|·|D|·N·H·T)	O(|w|·N·T)	−
DP-FedAvg [71]	O(|w|·|D|·N·H·T)	O(|w|·N·T)	O(|w|·N·T)
FedSDP [24]	O((|w|+d)·|D|·N·H·T)	O(|w|·N·T)	O((d·N·H+N)·T)
MSFL [61]	O(|w|·|D|·N·H·T)	O(|w|·N·T)	O((N+d)·N·T)
PBFL [27]	O(|w|·|D|·N·H·T)	$O(nd\log{\left(n\right)}^{2})$	$O(nd\log{\left(n\right)}^{3})$
PPAFL [18]	O(|w|·|D|·N·H·T)	O(|w|·N·T·M)	O(|w|·N·T)
RAFLS [34]	O(|w|·|D|·N·H·T)	O((|w|+d)·N·T)	O((|w|+d)·N·T)
Proposed	O(|w|·|D|·N·H·T)	O(|w|·N·T·logn)	O((|w|+|w|·log(n)+d)·m·N·T)

**Table 3:** Hyperparameter notations on MNIST.

Param	Value	Param	Value
N	20	M	10
*H*	20	batchsize	64
χ	{0, 0.2, 0.4}	λ	0.1
T	50	γ	0.08
α	0.3	ϵ	{0.5, 0.8, 1}
$\tau_{max}$	3	δ	$1\times 10^{-3}$

**Table 4:** Hyperparameter notations on CIFAR-10.

Param	Value	Param	Value
N	20	M	10
*H*	20	batchsize	32
χ	{0, 0.2, 0.4}	λ	0.03
T	50	γ	0.001
α	0.85	ϵ	{0.5, 0.8, 1}
$\tau_{max}$	3	δ	$1\times 10^{-3}$

**Table 5:** Hyperparameter notations on Heart Disease dataset.

Param	Value	Param	Value
N	20	M	10
*H*	20	batchsize	500
χ	{0, 0.2, 0.4}	λ	0.03
T	50	γ	0.01
α	0.85	ϵ	{0.5, 0.8, 1}
$\tau_{max}$	3	δ	$1\times 10^{-3}$

**Table 6:** Model accuracy.

χ=0, ϵ=1, $\delta =1\times 10^{-3}$, subsampling rate = 90%						
Dataset	FedAvg	DP-FedAvg	FedSDP	MSFL	RAFLS	Proposed
MNIST	93.01	92.46	87.66	92.46	90.07	92.77
CIFAR-10	77.61	77.70	55.98	75.51	75.25	77.75
Heart Disease Dataset	100	99.63	90.00	99.45	97.99	99.82

## Data Availability

Data are contained within the article.
